# Sputum cell‐free DNA: Valued surrogate sample for the detection of *EGFR* exon 20 p.T790M mutation in patients with advanced lung adenocarcinoma and acquired resistance to EGFR‐TKIs

**DOI:** 10.1002/cam4.3817

**Published:** 2021-05-01

**Authors:** Zheng Wang, Xiaoguang Li, Lin Zhang, Yan Xu, Mengzhao Wang, Li Liang, Peng Jiao, Yuanming Li, Shurong He, Jun Du, Lei He, Min Tang, Mingjun Sun, Li Yang, Jing Di, Guanshan Zhu, Lin Li, Dongge Liu

**Affiliations:** ^1^ Department of Pathology Beijing Hospital National Center of Gerontology Institute of Geriatric Medicine Chinese Academy of Medical Sciences Beijing P.R. China; ^2^ Department of Minimally Invasive Tumor Therapies Center Beijing Hospital National Center of Gerontology Institute of Geriatric Medicine Chinese Academy of Medical Sciences Beijing P.R. China; ^3^ Department of Respiratory and Critical Care Medicine Peking Union Medical College Hospital Chinese Academy of Medical Sciences Peking Union Medical College Beijing P.R. China; ^4^ Department of Cancer chemotherapy and Radiation sickness Peking University Third Hospital Beijing P.R. China; ^5^ Department of Thoracic Surgery Beijing Hospital National Center of Gerontology Institute of Geriatric Medicine Chinese Academy of Medical Sciences Beijing P.R. China; ^6^ Department of Medical Oncology Beijing Hospital National Center of Gerontology Institute of Geriatric Medicine Chinese Academy of Medical Sciences P.R. China; ^7^ Amoy Diagnostics Co., Ltd Xiamen P.R. China

**Keywords:** cell‐free DNA, EGFR‐TKI, liquid biopsy, lung adenocarcinoma, p.T790 M mutation, sputum

## Abstract

**Background:**

Sputum cell‐free DNA (cfDNA) is a valuable surrogate sample for assessing *EGFR*‐sensitizing mutations in patients with advanced lung adenocarcinoma. Detecting *EGFR* exon 20 p.T790 M (p.T790 M) is much more challenging due to its limited availability in tumor tissues. Exploring sputum cfDNA as an alternative for liquid‐based sample type in detecting p.T790 M requires potential improvement in clinical practice.

**Methods:**

A total of 34 patients with *EGFR*‐sensitive mutation‐positive lung adenocarcinoma and acquired resistance to the first generation of epidermal growth factor receptor‐tyrosine kinase inhibitors (EGFR‐TKIs) were enrolled. The sputum samples, and paired tumors and/or plasma samples were tested for p.T790 M mutation and concordance of p.T790 M status among the three sample types was analyzed.

**Results:**

The overall concordance rate of p.T790 M mutation between sputum cfDNA and tumor tissue samples was 85.7%, with a sensitivity of 66.7% and a specificity of 100%. The sensitivity for detecting p.T790 M in sputum cfDNA was 100%, 66.7%, and 0% in the three sputum groups of malignant, satisfactory but no malignant cells, and unsatisfactory, respectively. The combined results of plasma cfDNA testing and sputum cfDNA testing further increased the sensitivity to 100% for p.T790 M detection in satisfactory but no malignant cells sputum group.

**Conclusion:**

These findings revealed that cfDNA from malignant or satisfied but no malignant cells sputum is considered suitable for detecting p.T790 M mutation in patients with acquired resistance to first or second‐generation EGFR‐TKIs. The sputum cytological pathological evaluation‐guided sputum cfDNA testing assists in significantly improving the sensitivity of p.T790 M detection, bringing significant value for the maximal application of third‐generation EGFR‐TKIs in second‐line treatment.

## Graphical abstract

CfDNA from malignant or satisfied but no malignant cells sputum is suitable for detecting T790 M mutation in patients with acquired resistance to 1st or 2nd generation of EGFR‐TKIs. Sputum cytological pathological evaluation‐guided joint sputum and plasma cfDNA testing can significantly improve the sensitivity of T790 M detection, which brings significant value for maximal application of the third generation of EGFR‐TKI in second‐line treatment.

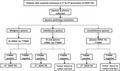

## 1 INTRODUCTION

The p.T790 M mutation accounted for 50–60% of patients with advanced lung adenocarcinoma (LUAD) and acquired resistance to first or second‐generation epidermal growth factor receptor‐tyrosine kinase inhibitors (EGFR‐TKIs).^1^ Osimertinib is a third‐generation tyrosine kinase inhibitor (TKI) that is designed to target p.T790 M displayed potent and durable clinical efficacy in second‐line treatment.^2^, ^3^ Thus, identification of p.T790 M is considered pivotal in patients with non‐small lung cancer (NSCLC).

Tissue or plasma‐based liquid biopsy is a routine approach used for detecting p.T790 M mutation in patients with acquired resistance to first or second‐generation EGFR‐TKIs.^4^, ^5^ Due to safety and increased complication risks during tumor tissue re‐biopsy, plasma‐based liquid biopsy is preferred.^6^ However, lower abundance of p.T790 M circulating tumor DNA (ctDNA) in plasma and spatiotemporal heterogeneity of ctDNA resulted in low sensitivity of plasma‐based p.T790 M detection, limiting its clinical application. The application of more sensitive techniques like the droplet digital PCR (ddPCR) and next‐generation sequencing (NGS) might improve the sensitivity of p.T790 M detection slightly, but still limited to the range of 60% to 70% when compared to tumor tissue testing.^7^, ^8^, ^9^, ^10^ Therefore, further efforts in improving the sensitivity of liquid‐based p.T790 M detection are still necessary for clinical practice.

Due to non‐invasive ability and easy to obtain in clinics, sputum has been used as one of the conventional cytological sample types for diagnostic purposes. In addition, the supernatant cell‐free DNA (cfDNA) of the sputum is verified to be suitable for *EGFR* sensitive mutation detection for treatment‐naïve patients with advanced NSCLC, and especially valuable when tumor tissue is not available.^11^ However, p.T790 M detection in patients with advanced NSCLC and acquired resistance to first or second‐generation EGFR‐TKIs requires elucidation. Hence, in this study, Super‐Amplification Refractory Mutation System (SuperARMS)^11^, ^12^ technology was used to analyze the mutation status of p.T790 M in sputum supernatant cfDNA. The concordances obtained among sputum, tumor, and plasma samples were analyzed.

## 2 MATERIALS AND METHODS

### 2.1 Study design

**FIGURE 1:**
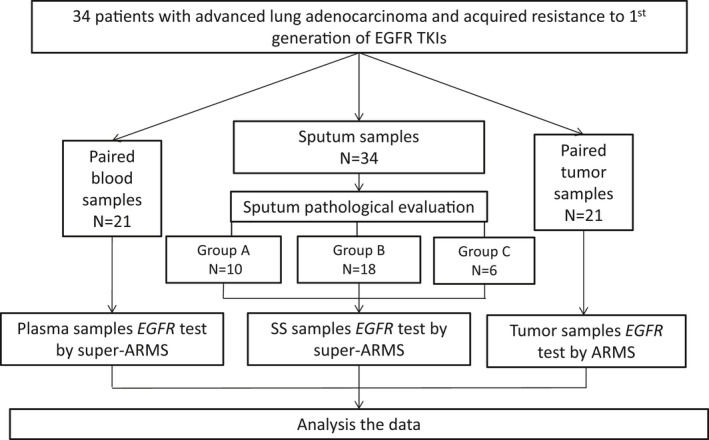
Flowchart of study design. Tumor samples: biopsy or non‐sputum cytological tumor samples; SS, Sputum supernatant; Group A: Malignant sputum; Group B: Satisfactory but non‐malignant sputum samples; Group C: Unsatisfactory sputum samples group

The sputum specimens were obtained from 34 patients with advanced LUAD who underwent treatment with first‐generation EGFR‐TKIs and acquired resistance from November 2018 to December 2019 in three medical centers of China (Beijing Hospital, Peking Union Medical College Hospital, and Peking University Third Hospital). All the sputum samples collected were expectorated. The patients with acquired resistance showed disease progression when on previous continuous treatment with an EGFR‐TKI according to the Response Evaluation Criteria in Solid Tumors1.1 (RECIST 1.1 criteria). Matched tumor samples (biopsy or non‐sputum cytology samples) or plasmas were collected simultaneously. All biopsy samples were collected under the guidance of computed tomography (CT) by percutaneous pulmonary puncturing of primary intra‐pulmonary tumor. The blood samples were simultaneously obtained or not more than 3 days post‐biopsy and the same for cytological tumor samples. The sputum specimens were stored at −80°C until use. Each sputum sample was evaluated by liquid‐based cytology. As shown in Figure 1, p.T790 M was detected and the performance was compared between sputum supernatant and matched tumor tissues/cells or plasma samples. This study was approved by the Beijing Hospital Institution Review Board (Approval number: 2020BJYYEC‐065–02).

### 2.2 Preparation of tumor tissue, cytological, and blood samples

The formalin‐fixed paraffin‐embedded (FFPE) tumor tissue and cytological blocks were prepared from 21 patients including 7 tumor biopsies and 14 cytological samples (10 malignant pleural effusion, 2 bronchoalveolar lavage fluid, and 2 cerebrospinal fluid). The tumor content and the presence of malignant cells were evaluated by Hematoxylin and Eosin (H&E) staining for each biopsy or cytology sample. The matched peripheral blood samples (10 ml each) were also collected from 21 patients above and centrifuged immediately. The plasma was then transferred into new tubes and stored at −80°C until use.

### 2.3 Sputum supernatants and sediments preparation

The mucolytic solution (MS) for sputum was prepared in a proportion of 9 ml of physiological saline solution and 1 ml of 0.5 mol/L of dithiothreitol (Sigma). The sputum specimen of 1–2 ml was taken and treated with 10 ml of MS. After vortexing and centrifuging, the supernatants were collected for cfDNA extraction and *EGFR* mutation detection, and the sediments were re‐suspended in PreservCyt Solution (Hologic Inc.) to make liquid‐based slides for pathological evaluation.^11^

### 2.4 Pathological evaluation of sputum, biopsy tumor tissues, and cytological specimens

Each liquid‐based slide of sputum specimens was evaluated by two cytologists (SRH and DGL) independently. The sputum specimens were classified into three categories according to the modified classification^13^: (1) Malignant: definite malignant cells were presented; (2) Satisfactory but no malignant cells: enough epithelial cells and macrophages, and no malignant cells were observed; and (3) Unsatisfactory: no or very fewer epithelial cells and macrophages were seen.

Biopsy tumor tissue and other cytological FFPE slides were reviewed by a pulmonary pathologist and a cytologist (ZW, SRH). More than 200 cancerous cells and tumor proportion of ≥10% on one slide were interpreted as satisfied tumor samples and used for *EGFR* mutation detection.

### 2.5 CfDNA and DNA extraction

Four milliliters of sputum supernatant and plasma were purified by centrifugation before cfDNA extraction. cfDNA was extracted according to the manufacturer's protocol (Amoy Diagnostics). The extracted DNA was eluted in 120 μl of DNA elution buffer and stored at −80°C until genotyping. Genomic DNA from tumor tissues or non‐sputum cytological FFPE blocks was extracted by an FFPE DNA Extraction Kit (Amoy Diagnostics) according to the manufacturer's protocol. The concentration of the purified cfDNA and genomic DNA was measured by the QuantiFluor^®^ dsDNA System^(a)^ (Promega, USA).

### 2.6 The p.T790 M detection

The p.T790 M in tumor tissues and non‐sputum cytological samples were detected using the ARMS *EGFR* Mutations Detection Kit (Amoy Diagnostics) according to the manufacturer's instructions. The p.T790 M in sputum supernatant cfDNA was analyzed by Super‐ARMS® *EGFR* Mutation Detection Kit^12^ (Amoy Diagnostics) according to the manufacturer's instructions. (Table S1.)

### 2.7 Statistical analysis

The data were analyzed by SAS software version 9.2 (SAS Institute Inc.). Chi‐square test was performed to detect any significant difference in the clinicopathological characteristics between the enrolled p.T790 M‐positive group and the p.T790 M‐negative group.

## 3 RESULTS

### 3.1 Patient characteristics

The baseline clinicopathological characteristics of 34 patients with advanced LUAD and acquired resistance to first‐generation EGFR‐TKIs enrolled in this study are shown in Table 1. No statistically significant differences were observed with regard to age, gender, smoking status, and types of paired samples between p.T790 M mutant and p.T790 M wild‐type patients. The median age of these patients was 64 years (range: 31–93). A total of 55.9% (*n* = 19 of 34) patients were females, which was slightly more than the number of males (44.1%, *n* = 15). Never‐smokers were dominant (64.7%, *n* = 22). Thirteen (38.2%) patients carried p.T790 M mutations in paired samples. The distribution of *EGFR* sensitive mutations included exon 19 deletion (52.9%, *n* = 18), exon 21 p.L858R (44.1%, *n* = 15) and exon 21 p.L861Q (2.9%, *n* = 1).

**TABLE 1:** Clinicopathological characteristics and p.T790 M status of patients with advanced lung adenocarcinoma and acquired resistance to first‐generation EGFR‐TKIs

	Overall (*n* = 34)	p.T790 M Mutation Status		
		p.T790 M positive (*n* = 13)	p.T790 M Negative (*n* = 21)	*P* value (95% CI)
Age				0.14(15.25–2.30)
Median (±SD)	64.0 ± 12.4	60.0 ± 12.5	66.5 ± 12.0	
Sex				1.0
Male	15(44.1%)	6(46.2%)	9(42.9%)	
Female	19(55.9%)	7(53.8%)	12(57.1%)	
Smoking status				0.46(0.45–0.47)
Never	22(64.7%)	7(53.8%)	15(69.6%)	
Former	12(35.3%)	6(46.2%)	6(30.4%)	
Paired samples				0.26(0.25–0.27)
Tumor	13(38.2%)	4(30.8%)	9(42.9%)	
Blood	13(38.2%)	4(30.8%)	9(42.9%)	
Both tumor and blood	8(23.5%)	5^a^(38.5%)	3(14.3%)	
Status of other EGFR mutations rather than p.T790 M in paired samples				0.89(0.89–0.90)
Exon 19 deletion	18(52.9%)	7(53.8%)	11(52.4%)	
Exon 21 p.L858R	15(44.1%)	6(46.2%)	9(42.9%)	
Exon 21 p.L861Q	1(2.9%)	0	1(4.8%)	
Sputum samples Cytological evaluation of the sputum samples				0.60(0.59–0.61)
Malignant	10(29.4%)	4(30.8%)	6(28.6%)	
Non‐malignant but satisfied	18(52.9%)	5(38.5.3%)	13(61.9%)	
Unsatisfied	6(17.6%)	4(30.8%)	2(9.5%)	

^a^ One of the five patients had tumor p.T790 M positive but blood p.T790 M negative, the remaining four patients had both tumor and blood p.T790 M positive.

### 3.2 p.T790 M mutation status in sputum supernatant cfDNA

The cfDNA extracted from the sputum supernatant was initially quantitated and qualified. The mean concentration of purified cfDNA was 16.87 ng/μl (0.89–24.56 ng/μl).

A total of 21 patients with paired tumor samples and sputum supernatant cfDNA samples were evaluable for p.T790 M mutation. The overall concordance rate of p.T790 M between sputum cfDNA and matched tumor samples was 85.7% (95% CI, 64.5%–95.9%), with a sensitivity of 66.7% (95% CI: 30.9%–90.9%) and specificity of 100% (95% CI: 69.9%–100%), see Table 2.

**TABLE 2:** Concordance analysis of p.T790 M mutation status between matched tumor samples and sputum cfDNA samples that are classified by cytological evaluation

Cytology evaluation		p.T790 M status in sputum cfDNA					
		Malignant *N* = 8		Satisfactory but no malignant cells *N* = 10		Unsatisfactory *N* = 3	
Matched tumor sample	p.T790 M	Positive	Negative	Positive	Negative	Positive	Negative
	Positive	4	0	2	1	0	2
	Negative	0	4	0	7	0	1
Sensitivity % (95% CI)		66.7% (30.9%, 90.9%)					
		100%		66.7%		0%	
		(39.6%, 100%)		(12.5%, 98.2%)		(0%, 80.2%)	
Specificity % (95% CI)		100% (69.9%, 100%)					
		100%		100%		100.00%	
		(39.6%, 100%)		(56.1%, 100%)		(5.5%, 100%)	
PPV % (95% CI)		100% (51.7%, 100%)					
		100%		100%		/	
		(39.6%, 100%)		(19.8%, 99.1%)			
NPV% (95% CI)		80% (51.4%, 94.7%)					
		100%		87.5%		33.3%	
		(39.6%, 100%)		(46.7%, 99.3%)		(1.8%, 87.5%)	

For sputum samples with the observation of malignant cells, both the sensitivity as well as the specificity of p.T790 M detection was 100% (95%CI: 39.6%–100%). For those samples without malignant cells but with sufficient epithelial cells and macrophages, the sensitivity and specificity of p.T790 M detection were 66.7% (95%CI: 12.5%–98.2%) and 100% (95%CI: 56.1%–100%), respectively. For unsatisfactory samples, the sensitivity and specificity of p.T790 M detection were 0% (95%CI: 0%–80.2%) and 100% (95%CI: 5.5%–100%), respectively.

### 3.3 Comparison of p.T790 M mutation status between sputum and plasma samples

In 21 of the 34 enrolled patients with paired plasma and sputum cfDNA samples, 8 were identified as p.T790 M positive in plasma samples, 4 patients were p.T790 M mutation positive in sputum sample only, and 9 patients were p.T790 M negative in both sample types, with an overall concordance rate between sputum cfDNA and matched plasma p.T790 M of 61.9% (95% CI, 40.8%–79.3%), (Table 3). In five patients with the observation of malignant cells in the sputum, four had p.T790 M positive in sputum supernatant cfDNA but only three of the four were positive in the plasma cfDNA. In 12 patients without malignant cells but with sufficient epithelial cells and macrophages in the sputum, p.T790 M was found to be positive in four sputum cfDNA samples and three plasma cfDNA samples with only one overlapping. In four patients with unsatisfactory sputum samples, p.T790 M was positive in two plasma cfDNA samples but none in the sputum cfDNA samples.

**TABLE 3:** Concordance analysis of p.T790 M status between matched plasma cfDNA and sputum cfDNA that are classified by cytological evaluation

Cytology evaluation		cfDNA isolated from sputum specimens tested by SuperARMS PCR					
		Malignant *N* = 5		Satisfactory but no malignant cells *N* = 12		Unsatisfactory *N* = 4	
Matched plasma sample	p.T790 M	Positive	Negative	Positive	Negative	Positive	Negative
	Positive	3	0	1	2	0	2
	Negative	1	1	3	6	0	2
Concordance rate % (95% CI)		80% (36.0%,98.0%)		58.3% (31.9%, 80.7%)		50% (15.0%,85.0%)	

In addition, in eight patients without malignant cells but with sufficient epithelial cells and macrophages, the sputum cfDNA samples were negative for p.T790 M, but two of the eight were p.T790 M positive in paired plasma cfDNA samples.

### 3.4 Joint testing of sputum and plasma samples improved the performance of cfDNA testing for p.T790 M in patients with satisfactory but no malignant cells sputum

Given that the sputum supernatant cfDNA p.T790 M detection was associated with sputum cytology evaluation, the performance of joint liquid biopsy for p.T790 M testing was analyzed according to the sputum cytological classification. For patients with malignant sputum, the sputum cfDNA sample was sufficient to detect p.T790 M, with both sensitivity and specificity of 100% (95%CI: 39.6%–100%). For patients with satisfactory but no malignant cells sputum, joint testing of both plasma cfDNA and sputum cfDNA reached p.T790 M detection sensitivity and specificity to 100% (95%CI: 31.0%–100%) as well. For patients with unsatisfactory sputum, the sputum cfDNA samples were considered not suitable for p.T790 M testing. Only one of the three patients had a plasma cfDNA sample, which was detected as p.T790 M positive, (Table 4).

**TABLE 4:** Overall concordance analysis of p.T790 M mutation status between liquid specimens and matched tumor samples that are classified by sputum cytological evaluation

Malignant sputum *N* = 8		Liquid specimens (sputum cfDNA)					
p.T790 M Mutation		Positive	Negative	Sensitivity % (95% CI)	Specificity % (95% CI)	PPV % (95% CI)	NPV% (95% CI)
Matched tumor sample	Positive	4	0	100% (39.6%, 100%)	100% (39.6%, 100%)	100% (39.6%, 100%)	100% (39.6%, 100%)
	Negative	0	4				
Satisfactory but no malignant cells sputum *N* = 10		Liquid specimens (sputum cfDNA +plasma cfDNA)					
p.T790 M Mutation		Positive	Negative	Sensitivity % (95% CI)	Specificity % (95% CI)	PPV % (95% CI)	NPV % (95% CI)
Matched tumor sample	Positive	3	0	100% (31.0%, 100%)	100% (56.1%, 100%)	100% (31.0%, 100%)	100% (56.1%, 100%)
	Negative	0	7				
Unsatisfactory sputum *N* = 3		Liquid specimens (plasma cfDNA)^a^					
p.T790 M Mutation		Positive	Negative	Sensitivity % (95% CI)	Specificity % (95% CI)	PPV % (95% CI)	NPV % (95% CI)
Matched tumor sample	Positive	1	1	50% (2.7%, 97.3%)	100% (5.5%, 100%)	100% (5.5%, 100%)	50% (2.7%, 97.3%)
	Negative	0	1				

^a^ The plasma samples of two cases were unavailable.

These data showed an improved performance in the detection of p.T790 M when applying sputum and plasma cfDNA samples jointly.

## 4 DISCUSSION

As reported, only about half of the patients with acquired resistance to first or second‐generation EGFR‐TKIs can accept re‐biopsy, and 30% of the re‐biopsied tumor tissues were found to be insufficient for downstream genotyping.^14^ Although the detection of p.T790 M occurs in more than 50% of patients with acquired resistance, it is of great importance in the clinical treatment decision. Therefore, alternative source of samples, especially liquid biopsy, are used in clinics for detecting p.T790 M. At present, plasma has been accepted as surrogate samples for p.T790 M mutation detection in clinical practices. Different technologies such as ddPCR and Cobas have been tried for plasma cfDNA p.T790 M detection, the outcomes had a sensitivity of around 60%, ^15^, ^16^ limiting its application in clinical practice. One of the key challenges in using plasma cfDNA for p.T790 M mutation detection is that the amount of ctDNA with p.T790 M and the proportion in total plasma cfDNA in some patients remained low, which was much less than *EGFR*‐sensitive mutations such as exon 21 p.L858R.^17^ Recent efforts have been made for more types of liquid biopsy samples including pleural effusion, urine, and sputum.^18^, ^19^, ^20^, ^21^, ^22^

Sputum has been the most commonly used sample for cytological diagnosis^23^, ^24^ as well as genotyping by its genomic DNA.^24^ Our recent study showed that the concentration of cfDNA from sputum is much higher than that from plasma and the cfDNA from the sputum supernatant has been proved to be a valuable surrogate sample for detecting *EGFR* sensitive mutations in advanced LUAD,^11^ which is encouraging for detecting p.T790 M using sputum cfDNA in patients with acquired resistance to EGFR‐TKIs. In the present study, 34 LUAD patients with acquired resistance to first‐generation EGFR TKIs were enrolled. All the samples detected with *EGFR* sensitive mutations. Compared with tumor samples, the concordance rate of detecting *EGFR* sensitive mutations in the sputum cfDNA were 100%, 40.0%, and 25.0% in three sputum groups of malignant, satisfactory but no malignant cells, and unsatisfactory, respectively. According to the current clinical guidelines,^25^, ^26^ the tissue sample is still regarded as the gold standard for p.T790 M detection, and liquid sample such as blood is the complementary sample type when the tissue sample is not available, as the clinical sensitivity of blood‐based p.T790 M detection is limited. Therefore, to evaluate the clinical value of testing sputum for p.T790 M, the tumor tissue sample was chosen for comparison rather than the blood sample. By a sensitive SuperARMS method, the detection of p.T790 M in the sputum cfDNA showed a specificity of 100% and overall sensitivity of 66.7%. When combined with cytological evaluation, compared to p.T790 M status in tumor samples, a sensitivity of 100% was achieved in testing the group of supernatant cfDNA samples with malignant sputum group, which was equivalent to what was observed in testing for sensitive *EGFR* mutations such as exon 21 p.L858R.^11^ A sensitivity of 66.7% was achieved in testing the group of supernatant cfDNA samples without malignant cells but satisfactory sputum group. p.T790 M was not detected in the group of supernatant cfDNA samples from unsatisfactory sputum. In the current study, the plasma sample from 21 of 34 enrolled patients was used. The concordance rate for detecting p.T790 M between sputum cfDNA and plasma cfDNA were 80%, 58.3%, and 50% in the three sputum groups of malignant, satisfactory but no malignant cells, and unsatisfactory, respectively. The discordance of p.T790 M testing results in the sputum and plasma samples in the group of patients without malignant cells but satisfactory sputum had potential complementary value for testing these two sample types simultaneously for detecting p.T790 M. The joint testing of both sputum cfDNA and plasma cfDNA significantly improved the sensitivity to 100% in this study.

Taking together with the results of our previous study,^11^ and with sputum cytology pathological evaluation as a quality control step, the preliminary data showed that the sputum cfDNA from patients with advanced NSCLC, either treatment‐naïve or acquired resistance, and with malignant sputum can be a surrogate sample, replacing tumor tissues, for detecting either *EGFR* sensitive mutations or p.T790 M mutation. For patients without malignant cells but satisfactory in sputum, join testing of sputum cfDNA and plasma cfDNA significantly improved the sensitivity of liquid sample‐based detection of p.T790 M. For patients with unsatisfactory sputum, the sputum cfDNA does not have any value in *EGFR* mutation detection, the plasma cfDNA instead might help to a limited extent.

**FIGURE 2:**
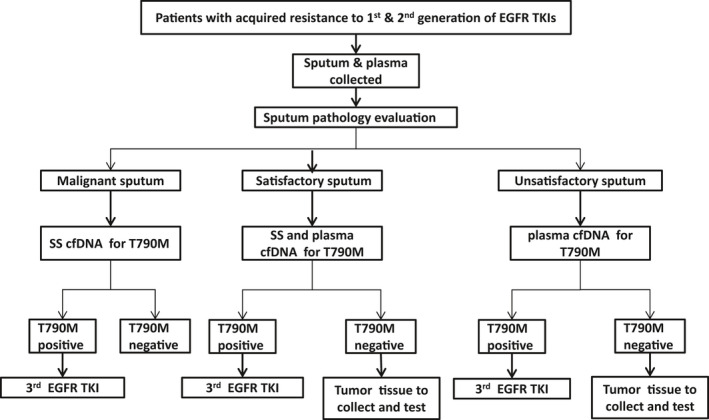
A stepwise algorithm for detecting p.T790 M mutation in patients with acquired resistance to first or second‐generation EGFR‐TKIs. Malignant sputum: malignant cells observed; Satisfactory sputum: enough epithelial cells and macrophages observed but no malignant cells; Unsatisfactory sputum: no or very fewer epithelial cells observed; SS, sputum supernatant

According to the preliminary results of the current study, a potential stepwise algorithm for p.T790 M mutation detection for patients with advanced NSCLC and acquired resistance to first or second‐generation EGFR‐TKIs, starting with sputum supernatant cfDNA testing (Figure 2): sputum sample and plasma samples are simultaneously collected, sputum sample pathological evaluated was proposed. The sputum cfDNA testing showed conclusive results for patients with malignant sputum; the sputum and plasma cfDNA would be considered suitable for p.T790 M testing for patients without malignant cells but satisfactory sputum; the plasma cfDNA directly was tested directly for patients with unsatisfactory sputum; the tumor tissue collection and detection should be tried for plasma cfDNA‐negative patients. Although the stepwise algorithm indicated that patients with sputum p.T790 M positive could be treated with third‐generation EGFR‐TKIs, sufficient support for the clinical data was still lacking. Therefore, the future clinical validation study is considered necessary.

However, there are several limitations in this study. Unfortunately, from our data, the difference in genetic mutations between sputum samples from patients with extrapulmonary and intrapulmonary lesions could not be implied. Theoretically, cfDNA can be obtained from all airway epithelial cells including tumor cells. Previous study reported no difference in sputum cfDNA from patients with centrally or peripherally located tumors during bronchoscopy.^27^ Thus, it could be expected that sputum cfDNA might overcome spatial heterogeneity in patients with multiple primary lung cancer lesions, while further investigation is definitely warranted. Negative result could be speculated from patients with only extrapulmonary recurrence lesions due to no cancer cell derived cfDNA in sputum. Therefore, sputum samples from patients with tumors in the extrapulmonary site might not be recommended for p. T790M test. Second, only SuperARMS assay was used for p.T790 M mutation detection in this study. There might be other sensitive technologies such as digital PCR or next‐generation sequencing that are suitable for testing sputum cfDNA to better detect p.T790 M mutation, which might be worthy to evaluate in future studies.^28^, ^29^, ^30^

In conclusion, this study preliminarily validated the feasibility of detecting p.T790 M using cfDNA isolation from the sputum supernatant in LUAD patients with acquired resistance to first‐generation EGFR‐TKIs. The sputum cytological pathological evaluation is considered vital for sputum supernatant sample genotyping. The malignant sputum supernatant samples can be used as surrogate samples for detecting p.T790 M mutations. Joint testing of both sputum cfDNA and plasma cfDNA can further improve the detection sensitivity in satisfied but no malignant cells sputum samples. A stepwise algorithm was proposed for detecting p.T790 M mutation in the present study. Further studies with larger sample size are suggested.

## DISCLOSURE

Guanshan Zhu is the stockholder of Amoy Diagnostics.

## ETHICS STATEMENT

This study was approved by the Beijing Hospital Institution Review Board (Approval number:2020BJYYEC‐065–02).

## Supporting information

Table‐S1

## Data Availability

Some or all data generated or used during the study are available from the corresponding author by request.
