# Supplementary material for: Sputum cell‐free DNA: Valued surrogate sample for the detection of EGFR exon 20 p.T790M mutation in patients with advanced lung adenocarcinoma and acquired resistance to EGFR‐TKIs
Source: Cancer Med. 2021 May 1;10(10):3323–31. doi: 10.1002/cam4.3817 (PMC8124129; doi:10.1002/cam4.3817)
Supplement: Supplementary file 1 — Table‐S1 [file CAM4-10-3323-s001.docx]

**Methods**

Preparation of blood samples

10ml EDTA anticoagulant tube was used to collect peripheral blood, and the blood should be separated within 2 hours. A two-step centrifugation was adopted for plasma separation, at 2000g for 10 minutes, then at 8000g for 10 minutes. Circulating free DNA was extracted following manufacturer’s protocol (Amoy Diagnostics, Xiamen, China).

The p.T790M detection

Amplification Refractory Mutation System (AMS)-PCR, which is based on oligonucleotides with a mismatched 3'-residue will not function as primers in the PCR under appropriate conditions^1^, was used for tumor mutant DNA detection in the present study. The analytical sensitivity and specificity of mutant DNA is 1% and 100%. Technically, FFPE samples can be detected with SuperARMS, but due to its high sensitivity, it may lead to false positive result due to damage or modification from formalin fixation to the tissue DNA.^2^

The SuperARMS is an upgraded technology with improved analytical sensitivity of 0.2-0.8% of mutant DNA in total DNA using SuperARMS to detection *EGFR* mutations in ctDNA, a clinical sensitivity (compared to that of matched tumor tissue) of greater than 80% has been achieved while maintaining high specificity. ^3^ Specific *EGFR* mutations detected by SuperARMS have been listed in Table S1.

In detail, SuperARMS PCR, utilized hairpin-structured primers replacing linear allele specific primer, and the spacer was used as a universal primer sequence. During amplification, the double-stranded binding portion of the primers was opened and combined with the target sequence to recognize targeted gene mutations. In addition, in the reaction system of SuperARMS, hot start DNA polymerase, with higher sensitivity and specificity, was used, and increasing the utility of samples. All these changes enhanced the discrimination ability of specific primers, and greatly improved the specificity and sensitivity.

Table S1. Details of the 41 *EGFR* mutations detected by SuperARMS and LOD for each *EGFR* mutation

| Exon | Mutation | Base Change | Cosmic ID | Name | %LOD |
| --- | --- | --- | --- | --- | --- |
| 18 | p.G719A | 2156G>C | 6239 | E-18-M1 | 0.20% |
|  | p.G719C | 2155G>T | 6253 | E-18-M3 | 0.40% |
| 19 | p.E746_A750del (1) | 2235_2249del15 | 6223 | E-19-M1 | 0.20% |
|  | p.E746_A750del (2) | 2236_2250del15 | 6225 | E-19-M2 | 0.20% |
|  | p.L747_P753>S | 2240_2257del18 | 12370 | E-19-M3 | 0.60% |
|  | p.E746_T751>I | 2235_2252>AAT  (complex) | 13551 | E-19-M4 | 0.40% |
|  | p.E746_T751del | 2236_2253del18 | 12728 | E-19-M5 | 0.40% |
|  | p.E746_T751>A | 2237_2251del15 | 12678 | E-19-M6 | 0.20% |
|  | p.E746_S752>A | 2237_2254del18 | 12367 | E-19-M7 | 0.20% |
|  | p.E746_S752>V | 2237_2255>T (complex) | 12384 | E-19-M8 | 0.20% |
|  | p.E746_S752>D | 2238_2255 del18 | 6220 | E-19-M9 | 0.40% |
|  | p.L747_A750>P | 2238_2248>GC (complex) | 12422 | E-19-M10 | 0.40% |
|  | p.L747_T751>Q | 2238_2252>GCA (complex) | 12419 | E-19-M11 | 0.20% |
|  | p.L747_E749del | 2239_2247del9 | 6218 | E-19-M12 | 0.40% |
|  | p.L747_T751del | 2239_2253del15 | 6254 | E-19-M13 | 0.40% |
|  | p.L747_S752del | 2239_2256del18 | 6255 | E-19-M14 | 0.40% |
|  | p.L747_A750>P | 2239_2248TTAAGAGAAG>C (complex) | 12382 | E-19-M15 | 0.40% |
|  | p.L747_P753>Q | 2239_2258>CA (complex) | 12387 | E-19-M16 | 0.40% |
|  | p.L747_T751>S | 2240_2251del12 | 6210 | E-19-M17 | 0.80% |
|  | p.L747_T751del | 2240_2254del15 | 12369 | E-19-M18 | 0.40% |
|  | p.L747_T751>P | 2239_2251>C (complex) | 12369 | E-19-M19 | 0.40% |
|  | p.L747_T751del | 2238_2252del15 | 23571 | E-19-M20 | 0.40% |
|  | p.L747_S752>Q | 2239_2256>CAA | 12403 | E-19-M21 | 0.20% |
|  | p.E746_T751>V | 2237_2252>T | 12386 | E-19-M22 | 0.60% |
|  | p.E746_T751>T | 2236_2253> ACG | / | E-19-M23 | 0.20% |
|  | p.L747_A750>P | 2239_2250>CCC | / | E-19-M24 | 0.40% |
|  | p.L747_K754>QL | 2239_2261>CAATT | / | E-19-M25 | 0.80% |
|  | p.E746_K754>EQHL | 2238_2261>GCAACATCT | / | E-19-M26 | 0.40% |
|  | p.E746_S752>EQ | 2238_2256>GCAA | / | E-19-M27 | 0.20% |
|  | p.E746_A750>QP | 2236_2248>CAAC | 13557 | E-19-M28 | 0.60% |
|  | p.E746_T751>Q | 2236_2253>CAA | 22999 | E-19-M29 | 0.40% |
| 20 | p.T790M | 2369C>T | 6240 | E-20-M1 | 0.20% |
|  | p.S768I | 2303G>T | 6241 | E-20-M2 | 0.20% |
|  | p.H773_V774insH | 2319_2320insCAC | 12377 | E-20-M3 | 0.40% |
|  | p.D770_N771insG | 2310_2311insGGT | 12378 | E-20-M4 | 0.60% |
|  | p.V769_D770insASp.VD770_N771insSVD | 2307_2308insgccagcgtg  2311_2312insGCGTGGAC | 12376  13428 | E-20-M5  E-20-M8 | 0.60%  0.40% |
|  | p.D770ASVD | 2309_2310AC>CCAGCGTGGAT | 13558 | E-20-M9 | 0.40% |
|  | p.H773_V774insNPH | 2319_2320insAACCCCCAC | 12381 | E-20-M10 | 0.80% |
| 21 | p.L858R | 2573T>G | 6224 | E-21-M1 | 0.20% |
|  | p.L861Q | 2582T>A | 6213 | E-21-M2 | 0.20% |

LOD: limit of detection

**References**

1 Newton CR, Graham A, Heptinstall LE, et al.: Analysis of any point mutation in DNA. The amplification refractory mutation system (ARMS). *Nucleic Acids Res* 1989; 17: 2503-2516.DOI: 10.1093/nar/17.7.2503.

2 Ye X, Zhu ZZ, Zhong L, et al.: High T790M detection rate in TKI-naive NSCLC with EGFR sensitive mutation: truth or artifact? *J Thorac Oncol* 2013; 8: 1118-1120.DOI: 10.1097/JTO.0b013e31829f691f.

3 Cui S, Ye L, Wang H, et al.: Use of SuperARMS EGFR Mutation Detection Kit to Detect EGFR in Plasma Cell-free DNA of Patients With Lung Adenocarcinoma. *Clin Lung Cancer* 2018; 19: e313-e322.DOI: 10.1016/j.cllc.2017.12.009.
